# Further exploration of the principles and mechanisms of brainwashing

**DOI:** 10.1073/pnas.2426192122

**Published:** 2025-05-23

**Authors:** Haitao Liu, Haogeng Sun, Yi Liu

**Affiliations:** ^a^Department of Neurosurgery, West China Hospital, Sichuan University, Chengdu, Sichuan 610041, China; ^b^West China Brain Research Centre, West China Hospital, Sichuan University, Chengdu, Sichuan 610041, China

In their article, Piantino et al. (1) report using gadolinium-based contrast-enhanced MRI to demonstrate that contrast-enhanced cerebrospinal fluid (CSF) enters the brain parenchyma through the MRI-visible perivascular space (MV-PVS). They assert that this is the first confirmation of MV-PVS as a critical factor in the distribution of CSF within the human brain. This finding marks the first visualization of the glymphatic system (GS) in humans, following its observation in mouse models in 2012 (2).

However, several unanswered questions remain regarding the results of this study. The first concern is that the study exclusively recruited patients with meningiomas, resulting in a small sample size. It’s still unclear whether this visualization results of the GS are specific to meningiomas. Thus, a larger sample size is needed for further experiments to confirm how reliable these results are. Furthermore, whether we’re talking about PVS or earlier studies on the GS, they’re more often found in neurodegenerative diseases like Alzheimer’s (AD) and Parkinson’s and can also show up in patients with cerebrovascular diseases and others (3–7). Thus, it is important to investigate whether this visualization process occurs in patients with other diseases, particularly AD, as the GS is thought to help clear tau and amyloid-β (Aβ) proteins, potentially aiding in the prevention and treatment of AD.

Second, according to the theory of brainwashing, the intracranial glymphatic-lymphatic circulation flows through the perivascular space network and ultimately returns to the meningeal lymphatic vessels (mLvs) (8). Therefore, as the visualization process in this paper, is it also possible to visualize the process of the GS returning from perivascular spaces to the mLvs? Visualizing this process may elevate our understanding of the brain’s waste clearance pathways to a new level.

Nonetheless, using neck lymphovenous anastomosis (LVA) to treat AD is becoming more popular, and some studies have shown that this type of surgery can help lighten the disease burden for AD patients (9, 10). Based on the principles of LVA, we’re also wondering whether LVA can help reduce the disease burden for AD patients by affecting the function of the GS and enhancing the clearance of proteins such as tau and Aβ and metabolic waste, but currently, there is no research to confirm this theory. The results of this study has achieved visualization of the GS, so can it help further refine the theoretical basis of the using LVA to treat AD and contribute to the diagnosis and treatment of diseases like AD?

In conclusion, this study really helps us understand the GS better. We look forward to seeing how these findings will impact future guidelines and improve patient outcomes.
